# Repeated storage of respired carbon in the equatorial Pacific Ocean over the last three glacial cycles

**DOI:** 10.1038/s41467-017-01938-x

**Published:** 2017-11-23

**Authors:** A. W. Jacobel, J. F. McManus, R. F. Anderson, G. Winckler

**Affiliations:** 1 Department of Earth and Environmental Sciences, Columbia University, New York, 10027 NY USA; 2 Lamont-Doherty Earth Observatory, Palisades, 10964 NY USA

## Abstract

As the largest reservoir of carbon exchanging with the atmosphere on glacial–interglacial timescales, the deep ocean has been implicated as the likely location of carbon sequestration during Pleistocene glaciations. Despite strong theoretical underpinning for this expectation, radiocarbon data on watermass ventilation ages conflict, and proxy interpretations disagree about the depth, origin and even existence of the respired carbon pool. Because any change in the storage of respiratory carbon is accompanied by corresponding changes in dissolved oxygen concentrations, proxy data reflecting oxygenation are valuable in addressing these apparent inconsistencies. Here, we present a record of redox-sensitive uranium from the central equatorial Pacific Ocean to identify intervals associated with respiratory carbon storage over the past 350 kyr, providing evidence for repeated carbon storage over the last three glacial cycles. We also synthesise our data with previous work and propose an internally consistent picture of glacial carbon storage and equatorial Pacific Ocean watermass structure.

## Abstract (web summary)

During glacial periods the oceans stored carbon removed from the atmosphere, yet identifying precisely where that storage occurred remains challenging. Here, the authors show that the deep equatorial Pacific Ocean was a reservoir for respired carbon during glacial periods for at least the last 350 kyr.

## Introduction

Glacial–interglacial climate cycles are well characterised in the late Pleistocene where records of atmospheric pCO_2_ and other greenhouse gases are available from gases trapped within ice cores^1^. These ice archives reveal pCO_2_ variations of 80–100 ppm on timescales of ~100 kyr with smaller but significant variance in the 41 and 23 kyr frequency bands. While several hypotheses have been proposed to explain the repeated glacial drawdown and deglacial release of atmospheric CO_2_, it is generally agreed that viable explanations must include carbon storage in the deep ocean^2–4^.

Key to answering questions about how and where marine carbon storage occurred during glacial periods is an understanding of deep ocean circulation, which, in combination with biological processes (productivity and respiration), is responsible for the distribution of CO_2_, O_2_, nutrients, and alkalinity in the ocean basins^5^. Studies aiming to quantify variability in these parameters on palaeo-timescales have emphasised the roles of the Southern Ocean^6–10^ and the deep Pacific Ocean^11–19^. The Antarctic Zone of the Southern Ocean is of particular interest because it is the place where the ‘biological pump’, which removes surface nutrients and carbon to depth, shows the greatest potential for increased efficiency relative to modern observations^7, 20^. The Pacific Ocean is of major significance because volumetrically it has the capacity to hold almost as much water and CO_2_ as all the other ocean basins combined and, due to the routing pathway of deep water, its northernmost reaches represent the oldest, most CO_2_ enriched waters in the ocean^21^.

Although there has been considerable dispute about the role of the Pacific Ocean in glacial carbon storage^17, 22^, previous work in the Subarctic and equatorial Pacific Ocean has been interpreted as showing evidence of reduced oxygen concentrations^12, 13, 15, 18, 19, 23, 24^, higher dissolved inorganic carbon (DIC) concentrations (lower δ^13^C)^23, 25–27^ and calcium carbonate dissolution (increased alkalinity)^11, 12, 23^ in concert with lower or similar local export productivity^12, 13, 28–32^ during the last glacial maximum (LGM) relative to Holocene levels. In concert, the data suggest that Pacific Ocean waters below ~2 km had higher concentrations of remineralised carbon during the last ice age^12, 24, 25, 27^, representing deep ocean sequestration of CO_2_ primarily due to changes in the efficiency of the upstream biological pump, and increased Antarctic water mass stratification, rather than local changes in productivity. These findings are consistent with the original ‘nutrient deepening hypothesis’^5, 33^ for enhanced oceanic carbon storage during the last ice age. They are also explained comprehensively by the ‘respired carbon deepening hypothesis’^12, 18, 24^, a modification that accounts for Cd/Ca proxy data showing relatively invariant deep water phosphate concentrations between the LGM and the Holocene in the deep Pacific^34^. The latter hypothesis^12^, proposes that during the last glacial period the ocean’s ‘biological pump’ was more efficient, particularly in deep water source regions, with more complete utilisation of macronutrients in the surface ocean contributing to lower concentrations of pre-formed nutrients in the deep sea. Reducing the inventory of pre-formed nutrients without changing total nutrient availability requires a corresponding rise in the inventory of regenerated nutrients, raising the respired CO_2_ concentration at depth, effectively sequestering carbon^4^. In contrast, during the Holocene, nutrient utilisation was less efficient, resulting in a reduction of respired carbon storage at depth, with the consequent transfer of carbon from the deep sea to the atmosphere.

The respired carbon deepening hypothesis combines Cd/Ca, nitrogen and carbon isotopes of foraminifera (δ^15^N and δ^13^C), authigenic uranium (aU), biogenic (excess) Ba flux (Ba_xs_) and opal production data from the last glacial period into an internally consistent framework, and in doing so poses important questions about deep ocean carbon sequestration on longer timescales. For instance: is deep ocean carbon storage a repeated feature of glacial periods? Does deep ocean oxygen limitation occur solely in concert with glacial maxima? How do millennial intervals of decreased atmospheric pCO_2_ such as Marine Isotope Stage 7d (MIS 7.4) manifest in records of deep ocean oxygenation?

**Fig. 1:**
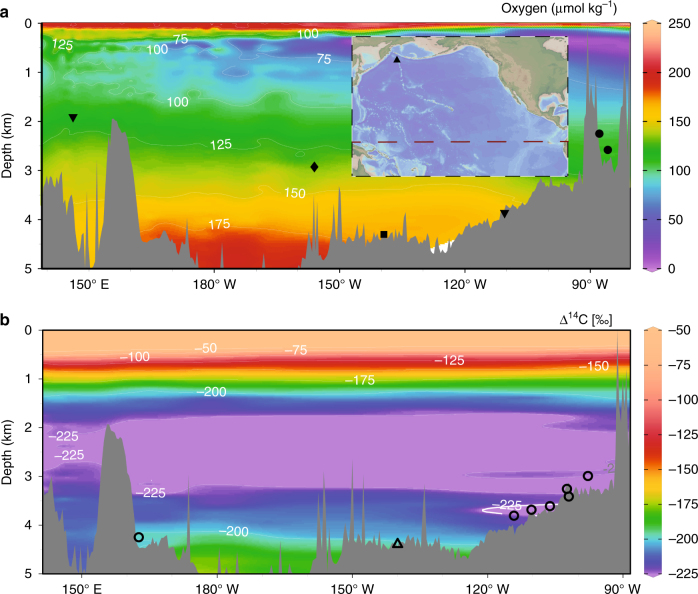
Maps of study area. Modern oxygen concentrations in μmol kg^−1^ ^21^ with the locations of Pacific Ocean records of bottom water oxygen as recorded by aU from ML1208-17PC (this study) (diamond), RC11-238 and RC13-140^13^ (dots), TT013-PC72^37, 38^ (square), ODP Site 849 and MD97-2138^13, 29^ (inverted triangles) and ODP Site 882^12^ (triangle) (inset only). Inset shows the approximate position of the oxygen concentration cross section in the context of the Pacific Ocean **a**. **b** Modern Δ^14^C concentrations^21^ with the location of radiocarbon data sets from TTN013-PC18^17^ (open triangle) and GS7202-15, PLDS 7G, VNTR01 10 GC, KNR73 3PC, KNR73 4PC, KNR73 6PG, and S67 15FFC^39^ (open circles). Note that the other ML1208 core sites discussed in this manuscript are at approximately the same longitude and depth as site 17PC. Basemaps made using Ocean Data View^72^

To address these questions, we reconstructed a record of U- and Th-series isotopes (^238^U, ^234^U, ^232^Th and ^230^Th) from RV Marcus G. Langseth cruise ML1208 sites 37BB, 31BB and 17PC in the Line Islands archipelago of the central equatorial Pacific Ocean (Fig. 1). The core sites range from 0.48° N to 7.0° N at ~160° W and sit at water depths around 3000 m (core locations in Supplementary Table 1). At present all three of these sites are bathed by North Pacific Deep Water (NPDW) with a local oxygen content of ~136 μmol  kg^−1^, phosphate concentration of ~2.6 μmol kg^−1^ and apparent oxygen utilisation of ~200 μmol  kg^-1^ ^21^. Surface waters at 17PC are derived from upwelling of the Equatorial Undercurrent (EUC) which originates from the mixing of South and North Pacific Subtropical Mode Water, Subtropical Cell Waters and Subantarctic Mode Water^35, 36^, cores 31BB and 37BB further to the north reflect a decreasing component of upwelled EUC water.

Here we present aU results from site 17PC spanning the last three glacial cycles at millennial resolution and aU results from sites 31BB and 37BB over the last glacial cycle. Our reconstructions expand the published aU data coverage to include the central equatorial Pacific Ocean and shows NPDW had significantly lower oxygen concentrations than at present during MIS 2, 4, 6 and 8. We also provide a perspective on watermass structure to reconcile our results with data interpreted as suggestive of no increase in Pacific Ocean storage of respired carbon during the LGM^17^, previously inconclusive data on bottom water oxygen state from 140° W^37, 38^ and results taken to indicate that Lower Circumpolar Deep Water was the sink for respired carbon^39^. Finally, we discuss our reconstruction of deep water oxygen history in the context of changes in global climate and pCO_2_. Our results provide evidence that the deep Pacific Ocean was an active reservoir for respired carbon during glacial periods of at least the last 350 kyr.

## Results

### Authigenic uranium

**Fig. 2:**
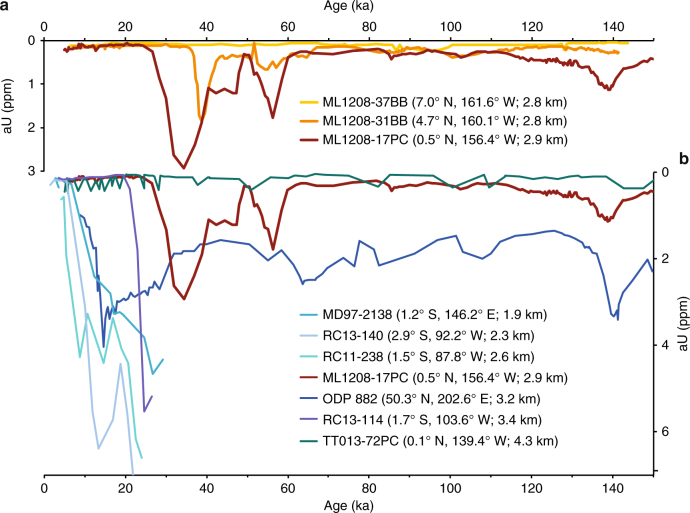
Records of aU from the Pacific Ocean. Sedimentary aU records from ML1208 sediment cores 17PC (dark red), 31BB (orange) and 37BB (gold). **a**. Comparison of aU records from the Pacific Ocean including 17PC (this study), ODP Site 882^12^, TT013-72PC^37, 38^, MD97-2138^13^, RC11-238^13^ and RC13-140^13^ with colours and locations as depicted in **b**

**Fig. 3:**
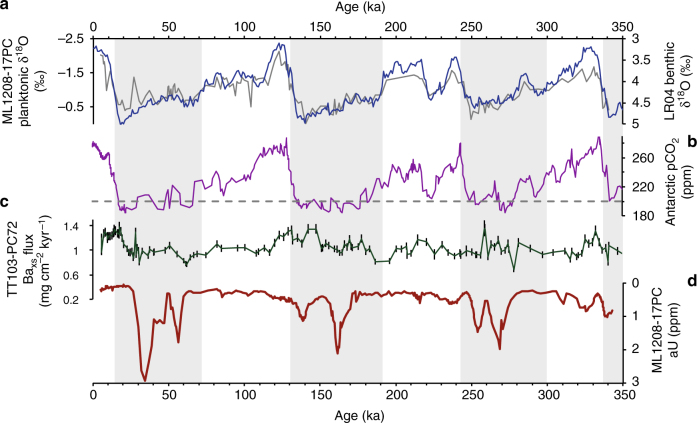
Climate and aU. Benthic oxygen isotope stack (blue)^61^ and local planktonic oxygen isotope stack from ML1208-17PC (grey) (this study) **a**. Composite of Antarctic pCO_2_ records^73.^ **b**. Central equatorial Pacific Ocean (TT013-PC72) excess barium fluxes (dark green) with 1*σ* error bars^32.^ **c**. Authigenic uranium concentrations from site 17PC in the central equatorial Pacific Ocean (note the inverted *y* axis) with average 1*σ* error bar indicated. **d**. All Antarctic records are on the AICC2012 age model^74^ and all other records are as originally published. Grey bars indicate glacial climate periods

Records of aU were derived for cores 17PC, 31BB and 37BB (Figs. 2 and 3) on the basis of measured ^238^U and ^232^Th activities. Uranium is a redox-sensitive metal and is present in oxygenated seawater as soluble uranyl carbonate^40^. At the seafloor, inorganic precipitation of U may occur within the porewater of sediments under conditions where the combination of low bottom water oxygen concentrations together with in situ respiration drive the sediments to anoxia and to a redox state of iron reduction^41–46^. The record of aU from site 17PC shows variability in the range of 0–0.5 ppm with periodic, abrupt increases in aU reaching values of 2–3 ppm. Large (>1 ppm) peaks of aU are observed at 34.4, 56.3, 138.8, 161.4, 254.2 and 268.6 ka, appearing during minima in pCO_2_ concentrations, and intervals of increased northern hemisphere glaciation as recorded by heavy oxygen isotope excursions in the planktonic isotope record from Site 17PC and the LR04 benthic foraminifera stack (Fig. 3). Site 37BB shows a more muted pattern of variability relative to site 17PC, with aU values peaking around 2 ppm. The maximum aU concentration occurs at 38.7 ka and a broad increase in values is also observed around 55 ka. These elevations in aU abundance are superimposed on a record which otherwise shows little variability with values of ~0.5 ppm. In contrast with cores 17PC and 31BB, the record from site 37BB displays no obvious peaks in aU abundance, consistently reflecting aU concentrations below 0.5 ppm over the interval studied. The maximum aU concentrations reported here are within the range of those observed during the LGM at sites from the eastern equatorial Pacific Ocean (west of 83.6° W)^13, 47, 48^ and subarctic North Pacific^12^, but are approximately an order of magnitude higher than maximum concentrations measured at TT013-PC72 (0.1° N, 139.4° W; 4300 m depth) (hereafter PC72)^37, 38^ (Fig. 2b), <20° (2220 km) east of our site. We reconcile these differences in the following discussion of palaeo watermass geometry.

## Discussion

Concentrations of aU in cores 17PC and 31BB are substantially greater than would be expected if the uranium present was derived solely from uranium incorporation into foraminiferal calcite or organic material (Methods) (Fig. 2). Instead, we propose that the primary determinant of aU abundance at sites 17PC and 31BB is the redox state of the sedimentary environment. Thus, the precipitation of aU at these two sites was (and is) likely controlled by the combined influence of bottom water oxygen supply and the rate of oxygen consumption, as determined by the respiration of organic matter. Accordingly, the precipitation of aU may either have been driven by a decrease in the supply of oxygen from bottom waters, or by an increase in respiration due to a larger flux of organic material from the overlying surface waters. The abyssal waters bathing sites 17PC and 31BB (NPDW) were most recently at the surface in the Southern Ocean, and therefore any decrease in ventilation rate or increases in respiration along the flow pathway could reduce oxygen delivery to the central equatorial Pacific Ocean. Alternatively, aU precipitation at these sites might be favoured if the rain of organic material and associated respiration at depth increased locally during glacial periods, with no change in ventilation required. Here we investigate these two possibilities.

Abundant proxy data including opal fluxes, excess barium fluxes, ^231^Pa/^230^Th ratios and the δ^15^N of planktonic foraminifera characterise the last glacial period as having similar or lower export productivity than during the Holocene interglacial at 160° W in the central equatorial Pacific^31, 75^, as well as further to the west^29^ and much of the east^32, 75^. In the absence of increased biogenic fluxes, higher aU abundance during the last glacial period must therefore be controlled primarily by decreased bottom water oxygen concentrations. This decreased [O_2_], in combination with respiration at a relatively invariant background level, lowered the redox state of porewaters to the point of iron reduction, allowing for the precipitation of aU.

Further evidence for deep ocean oxygen changes as the crucial control of aU precipitation during the last glacial period comes from a comparison of the record of 17PC to the other ML1208 sediment cores. Figure 2a illustrates that the amount of aU in the sediments decreases northwards away from the equator (from 17PC to 31BB to 37BB). Bottom water oxygen levels are likely to have changed synchronously at these ML1208 sites because of their close proximity in space and depth, so we attribute the northward decrease in aU to the corresponding decrease in organic carbon rain, decreasing respiration in the sediments. This inference is consistent with the decreasing export of organic carbon with increasing distance from the equator, as expected due to depletion of nutrients as waters mix poleward from their upwelling source at the equator. Indeed, site 37BB appears to have such a low organic export flux that conditions for aU precipitation are not reached over at least the last 150 kyr. This comparison suggests that a threshold level of organic carbon respiration is a necessary but insufficient condition for aU precipitation.

At present no flux-normalised data on export productivity extending beyond the LGM are available from the Line Islands region at 160° W. Fortunately, flux-normalised records of barium excess, representing organic carbon export, exist from site PC72 at 140° W^32^. Because surface productivity at site PC72 is also fed by the EUC, surface waters at 17PC and PC72 have zonally homogenous nutrient concentrations^32, 49^. We observe little difference between the present day nutrient profiles of water upwelled at the two sites^49^ and expect any past changes in the properties of EUC source waters to have affected both sites similarly.

Productivity data from site PC72 (Fig. 3c) confirm the observation from the Line Islands area that productivity during the last glacial and LGM was lower in the central equatorial Pacific Ocean relative to Holocene levels. Generally, the record shows relatively little variability between glacial and interglacial periods. Indeed, late MIS 6 and early MIS 5 export fluxes appear similar, as do those during late MIS 8 and MIS 7. Most importantly, there are no detectable increases in productivity coincident with increases in aU. The lack of concordance between observed records of EUC-driven export production in comparison with aU indicates local changes in organic carbon export were not the primary factor regulating aU precipitation during glacial periods. Instead, we infer that peaks of aU precipitation during the last three glacial cycles resulted from a decrease in advected bottom water oxygen concentrations which brought post-respiration porewater redox levels below the threshold for aU precipitation.

In contrast with other records from the Pacific Ocean basin which show maximum aU fluxes between 27 and 15 ka^12, 13^ (Fig. 2b), our record from 17PC in the central equatorial Pacific Ocean shows a peak of uranium precipitation nominally occurring at ~34 ka. While the late aU peak at ODP Site 882 in the Subarctic North Pacific Ocean can likely be attributed to a late deglacial productivity pulse^12, 19^, other records from the equatorial Pacific Ocean (MD97-2138, RC11-238 and RC13-140)^13^ suggest aU precipitation was ongoing at these sites until late in the deglaciation (Fig. 2b). Although it is possible that redox conditions were spatially and temporarily variable across the glacial equatorial Pacific Ocean, we propose the difference is more likely due to post-depositional alteration (burndown) of the Line Islands records at 17PC and 31BB after the LGM.

Diagenetic alteration of aU down core may occur in sediments following an increase in oxygen at the sediment–water interface, as would occur with reinvigoration of deep water ventilation. As oxygen diffuses into porewaters it creates a front where organic carbon stock is oxidised and aU is dissolved into solution^50^. Once dissolved, aU may either be lost to seawater or diffuse deeper into the sediment where anoxic conditions persist and the U can be re-precipitated. We suggest that during the Holocene, oxygenation of bottom waters caused the removal of aU as much as 50 cm down core at site 17PC. This magnitude of oxygen penetration is well within the observed range of sedimentary oxygen diffusion^15^ and would account for the temporal difference between the aU peak in the record from 17PC and those records from the eastern and western equatorial Pacific^13^, where the much larger sediment accumulation rates limit the time interval over which burndown may have affected the aU record.

Further evidence that the timing of the latest MIS 2 peak in 17PC represents loss of aU comes from comparing the timing of corresponding peaks in cores 17PC and 31BB (Fig. 2a). The LGM aU peak at 31BB appears to occur before that at 17PC (~40 ka vs. ~34 ka), as might be expected if Holocene oxidation of LGM-deposited organic carbon occurred at approximately the same magnitude at both sites. Since 31BB has a lower organic carbon rain (due to its off equator position), post transition re-oxidation would occur at a greater rate and to a deeper depth (and correspondingly older age) at site 31BB relative to site 17PC. This expectation matches our observations, suggesting that the timing of the latest MIS 2 peaks in both 31BB and 17PC are a consequence of post-depositional alteration of the aU signal. We suggest that post-depositional re-oxidation also explains the absence of an aU peak in 31BB during the latest MIS 6, at a time when PC17 preserved a peak of aU. Given the lower productivity at site 31BB the original deposition of aU is unlikely to have been as significant or as long-lived as at 17PC. Since its lower organic carbon rain also makes it susceptible to greater burndown, the absence of a peak at 31BB is not unexpected.

Indications of down core aU mobilisation during the LGM portion of our record raise the question of whether the double peaks observed within MIS 2, 6 and 8 (specifically the early glacial peaks) might be related to post-depositional sedimentary processes. Based on the abundance of the ^238^U-decay chain daughter isotope ^230^Th we provide evidence that this is not the case. Instead, we suggest both early and late glacial aU peaks are original to the depth intervals in which they are measured and if anything, represent peaks of a smaller magnitude than those originally precipitated in situ.

**Fig. 4:**
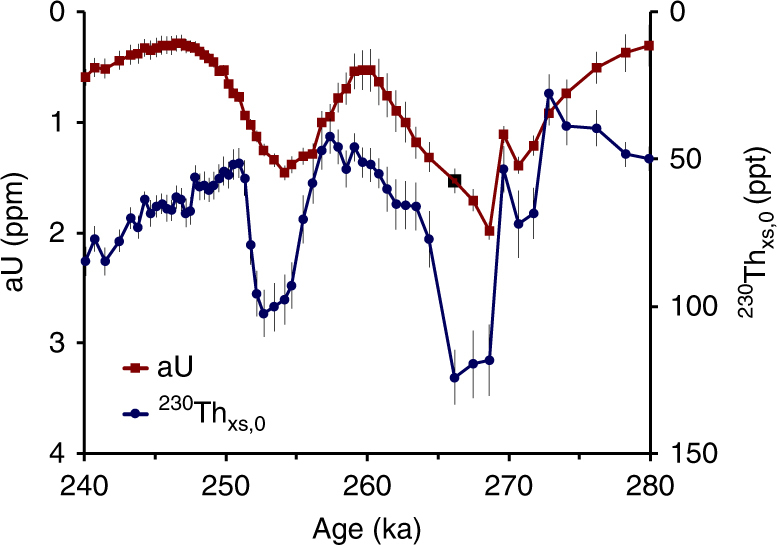
Co-variation of ^230^Th_xs,0_ and aU. Records of aU (red) and ^230^Th_xs,0_ (blue) from site 17PC over MIS 8. Error bars represent 1 standard deviation uncertainties

Examination of U and Th isotope abundances in 17PC reveals that the apparent ^230^Th_xs,0_ concentrations show an unexpected, positive correlation with authigenic ^238^U (see ‘Methods’—Use of ^230^Th_xs,0_). We illustrate this relationship in detail using the example of MIS 8 (Fig. 4) but note that the same relationship holds for all aU peaks except the most recent LGM interval (in which it may still be developing) (Supplementary Fig. 2). The association of high ^230^Th_xs,0_ and authigenic ^238^U could be due to very low particle accumulation rates coinciding with aU deposition. An alternative explanation, one which we consider more likely, is that ^238^U was oxidised and subsequently lost to the water column sometime after authigenic precipitation. Post depositional loss of ^238^U would lead to the ‘abandonment’ of its daughter isotope, causing the appearance of anomalously high ^230^Th_xs,0_ concentrations. Given that aU is readily mobilised to porewaters upon oxidation^51^ and can be lost to the water column^50^, we propose that high ^230^Th_xs,0_ concentrations reflect post-depositional oxidation and removal of aU 10–20 kyr after initial precipitation (Supplementary Notes 1 and 2, and Supplementary Fig. 3). This duration of ^238^U decay prior to dissolution would be consistent with either a temporary increase in the oxygen content of bottom waters between periods of low oxygen, or deglacial ventilation of deep waters. This pattern of pulsed oxygen limitation during glacial periods has not been previously observed, possibly due to limited data resolution and is a feature worthy of future work. In summary, unusually high ^230^Th_xs,0_ concentrations indicate that not only does the aU in core 17PC reflect original deposition in situ, but it is likely that the initial aU concentration was actually higher than observed at present due to some intervening loss to seawater.

A substantial body of research has focused on evaluating the evidence for a glacial reservoir of carbon in the deep Pacific Ocean and on defining its extent. Here, we integrate our new data with existing research to propose a picture of glacial watermass stratification reconciling apparently inconsistent data from the deep central^11, 17, 38^ and eastern Pacific^15, 39^.

At present, the deep equatorial Pacific Ocean is occupied by two main watermasses, NPDW and Antarctic Bottom Water (AABW). Southward flowing NPDW occupies depths between ~2 and 4 km while the deepest Pacific Ocean is bathed by northward flowing AABW. As might be predicted given the flow pathways of these two watermasses, NPDW is relatively older and less oxygenated than the underlying AABW (Fig. 1). Last glacial maximum grain size data from the North Chatham drift in the southern Pacific Ocean indicate increased formation of AABW and inflow to the Pacific^52^. Although the fraction of North Atlantic Deep Water in AABW was likely different during the LGM, stronger AABW flow would have provided a source of relatively more recently ventilated waters to the deepest Pacific Ocean, similar to the present configuration. This interpretation is supported by records of aU from sites PC72^37, 38^ (Fig. 2b) and ODP Site 849^29^ (not shown). These deep Pacific Ocean sites (at 4.3 km and 3.8 km, respectively) demonstrate neither the concentration nor structure of aU observed in other records from the Pacific Ocean. Whatever the oxygen concentration in northward flowing bottom waters during glacial periods, if glacial circulation followed a pattern similar to that which exists today then the apparent inconsistency between the deepest Pacific Ocean records of aU accumulation (PC72 and ODP Site 849) and our work (supported by the work of Bradtmiller et al.^13^ and Mills et al.^15^) could be easily understood as the result of sampling sites bathed by different watermasses. This hypothesis is supported by LGM radiocarbon data from the eastern equatorial Pacific^39^, central equatorial Pacific^17^ and south Pacific^53^ which together indicate that the watermass below ~3.5 km (3.8 km in the eastern equatorial Pacific Ocean) was more recently ventilated (younger) than the overlying watermass. Specifically, a stratification structure similar to present day would readily explain the absence of a significantly different surface to deep (~4.4 km) radiocarbon gradient at the LGM relative to present^17^.

The majority of existing estimates for the upper bound of glacial NPDW come from North Pacific Ocean δ^13^C data and indicate a watermass high in respired carbon (low δ^13^C) as shallow as 2 km^25, 27^. In the equatorial Pacific Ocean the estimates for LGM NPDW are from 1.9^13^ to 2 km^25^ in the western portion of the basin and 2^54^ to 2.2 km^13^ in the east. Combining these estimates of the bounds of NPDW suggests the LGM watermass may not have occupied a significantly different depth range than at present. Instead, the more significant glacial change appears to have been to the watermass properties.

The observation of repeated aU maxima during the last three glacial periods in the absence of concurrent increases in organic carbon fluxes provides evidence that respired carbon storage in the deep Pacific Ocean has been a recurrent feature of at least the last three ice ages. The timing of these aU increases indicates that the deep ocean may have been periodically oxygen-limited during glacial periods as a consequence of increased carbon storage and decreased rates of ocean ventilation. These findings are consistent with those from a site on the East Pacific Rise^15^, but our proxy appears to be a uniquely sensitive measure of deep water conditions as aU documents not just one, but multiple intervals of low oxygen during each of the last three glacial periods. These results suggest that geochemical conditions conducive to authigenic uranium deposition in Pacific Ocean sediments at ~3 km water depth were closely associated with an atmospheric pCO_2_ concentration of ~200 ppm (Fig. 3b) with some range in sensitivity attributable to the variable influence of marine organic productivity and the biogeochemical activity of the terrestrial biosphere^55^. Evidence for a relationship between 200 ppm pCO_2_ and aU precipitation raises the question of what bottom water oxygen concentration is related to this apparent threshold. Furthermore, the observation raises the possibility that brief intervals of low oxygen abundance (for example MIS 7d) are not well preserved in the core due to post-precipitation oxidation. Thus, while we conclusively identify the existence of an oxygen-poor, carbon-rich watermass in the Pacific Ocean, future work to quantitatively determine the oxygen content (as previously done for the Atlantic^56–58^) is essential for determining the precise timing and spatio-temporal extent of respiratory carbon storage.

Conclusions drawn from our new central equatorial Pacific Ocean records of sedimentary authigenic U are in agreement with previous studies of aU covering the last ~20^13^ and ~150 kyr^12, 18^, indicating that the Pacific Ocean stored a large volume of respired carbon during the last glacial period. These independent lines of evidence conclusively identify vast areas of the deep Pacific Ocean, including the central (this study), east^13, 15^, west^13^, north^12, 18, 19, 23, 24^ and south^53^ Pacific Ocean, as reservoirs for respired carbon during the last three glacial cycles. In contrast with some previous work^17, 39^, we infer a ‘floating’ pool of respired carbon between 2 and 3.5 km depth in the central equatorial Pacific Ocean and conclude that existing proxy data reflecting LGM deep water circulation and carbon storage can be reconciled without invoking a significantly different glacial watermass structure in the central equatorial Pacific Ocean. Our records thus highlight the importance of the deep Pacific Ocean as a reservoir for atmospheric pCO_2_ storage during glacial periods, and suggest that processes responsible for increasing the carbon inventory of the basin were active components of the climate system over at least the last 350 kyr.

## Methods

### Study site

This study presents records of U- and Th-series isotopes (^238^U, ^234^U, ^232^Th and ^230^Th) reconstructed from sediment cores ML1208-17PC, ML1208-31BB and ML1208-37BB collected from the Line Islands archipelago of the central equatorial Pacific Ocean (Fig. 1). Sediments at all three sites are foraminifera oozes with low organic and terrigenous components.

Age–depth relationships for these three cores have been previously published to 150 kyr. The age model for core 17PC beyond 150 kyr is constrained in the same way as the earlier part of the record, using δ^18^O isotope ratio constraints from the planktonic foraminifera *Globigerinoides ruber* ^59^ and previously published radiocarbon dates^60^. The isotope stratigraphy for 17PC was tuned to the LR04 benthic stack^61^ using a Monte-Carlo-enabled cross-correlation maximisation scheme coupled with a random walk algorithm known as MonteXCM^62^. Age–depth control points specified by MonteXCM were refined in Bchronology^63^ to better constrain age uncertainties, which are 2.3 ka (1*σ*). The resulting apparent sedimentation rates at site 17PC average 2.1 cm ka^−1^ (Supplementary Fig. 1). The age models employed here are consistent with the most recently published versions for 17PC, 31BB and 37BB^60^ but vary slightly from those originally published^59^ due to improved age control.

### Radiogenic isotope measurements

New data presented in this study are 814 uranium isotope measurements from cores ML1208-17PC, 31BB and 37BB analysed by isotope dilution on an Element 2 ICP-MS at the Lamont–Doherty Earth Observatory of Columbia University. For each depth interval ~200 mg of sediment was spiked with ^236^U and ^229^Th prior to dissolution and digestion using HNO_3_, HF and HClO_4_, following methods outlined in ref. ^64^. The U and Th fractions were concentrated using anion-exchange column chemistry following known adsorption behaviours^65^. An internal standard with similar chemical and physical properties to the ML1208 sites was evaluated with each batch of samples to determine data reproducibility with resulting relative standard deviations of 2.7%, 2.3% and 3.7% for ^238^U, ^230^Th and ^232^Th, respectively. Background levels of U and Th were evaluated for each batch using blanks processed alongside samples but containing no sediment. Results show blanks for all isotopes at <1.5% of even the lowest sample values. We report uncertainties calculated for each data point at the 1*σ* level, including the propagated uncertainty due to counting statistics, the mass bias correction, counting gain, spike measurements and the fraction of the sediment derived from lithogenic source material^13, 66, 67^. The location and depth information for all cores mentioned in this study can be found in Supplementary Table 1.

### Authigenic uranium proxy systematics

Uranium is a redox-sensitive metal and is present in oxygenated seawater as soluble uranyl carbonate^40^. In the upper water column, particulate U is generally associated with either detrital silicates or with organic carbon^68^, although a small amount of U (~0.012–0.036 ppm) can be incorporated into the calcite tests of foraminifera^69^. As particles descend through the open ocean water column most U precipitated in conjunction with C_org_ is remineralised, although this recycling process appears to be less efficient at the ocean margin where dissolved oxygen concentrations can be much lower than in typical open ocean settings^41, 68^. At the seafloor, two more processes may contribute U to the sediments. First, inorganic precipitation of U may occur within the porewater of sediments under conditions of complete anoxia accompanied by iron reduction. This process occurs due to the reduction of U(VI) to U(IV) by iron reducing bacteria^70^. As the precipitation of reduced uranium proceeds, a concentration gradient may form between the high [U] seawater and low [U] porewater, creating a diffusive flux into the sediment that can generate intervals of high U^12, 13, 43^. Authigenic precipitation in porewaters is thought to be the most important (~75%) mechanism of U removal in the ocean^43, 44^. A second mechanism of sedimentary U addition at the seafloor is precipitation in association with hydrothermal vent systems^15^. These processes scavenge U from seawater and constitute a significant global sink of U, although the impact of hydrothermal removal is restricted to near-ridge environs and does not influence the study site addressed here.

Despite the geochemical complexity of U deposition in sediments, U is typically characterised as either detrital/lithogenic (of terrestrial origin) or authigenic (of marine origin). In the central equatorial Pacific lithogenic U is transported via the same aeolian processes delivering ^232^Th and thus can be constrained using the empirically determined activity ratio between lithogenic or detrital ^238^U and ^232^Th (0.7 ± 0.1) for the Pacific^71^ such that ^238^U_detrital_ = 0.7* ^232^Th_meas_, and all additional U not lithogenic in origin is considered to be authigenic.

### Use of ^230^Th

Thorium-230 may be introduced to the sediment in two ways: via the decay of particulate uranium (of either detrital or authigenic origin), or via scavenging of ^230^Th onto particles as they descend through the water column. The former ^230^Th (ingrowth from radioactive decay) is referred to as supported ^230^Th, whereas the latter is considered unsupported or excess (xs). Excess ^230^Th is decay corrected to quantify the initial ^230^Th_xs_ (^230^Th_xs,0_). Changes in the concentration of ^230^Th_xs,0_ are primarily a function of dilution by other sedimentary constituents (e.g.: organic matter, lithogenic particles and calcium carbonate).

### Data availability

Line Islands U and Th data will be archived at the National Oceanic and Atmospheric Administration National Centers for Environmental Information (NCEI) database upon publication and are also available as a supplement to this manuscript.

## Electronic supplementary material

Supplementary Information 1

Description of Additional Supplementary Files

Dataset 1
