# Supplementary material for: Repeated storage of respired carbon in the equatorial Pacific Ocean over the last three glacial cycles
Source: Nat Commun. 2017 Nov 23;8:1727. doi: 10.1038/s41467-017-01938-x (PMC5700088; doi:10.1038/s41467-017-01938-x)
Supplement: Supplementary file 1 — Supplementary Information 1 [file 41467_2017_1938_MOESM1_ESM.pdf]

Supplementary Note 1: Thorium excess and relationship to authigenic uranium

We propose that high $^{230}Th_{xs,0}$ concentrations coincident with aU peaks reflect post-depositional oxidation and removal of aU 10-20 kyr after its initial authigenic precipitation. This hypothesis is supported by the observation of ongoing oxidation of the LGM aU peak (a process similar to that which likely affected the relict aU peaks), and the observation that bottom waters became re-oxygenated during periods of increased ventilation following the deposition of the glacial aU peaks. Our hypothesis of post emplacement ^{238}U dissolution and ^{230}Th daughter isotope 'abandonment' finds additional support in a modeling experiment designed to verify a range of these dissolution scenarios. We evaluate these scenarios by assessing the changing correlation between aU and $^{230}Th_{xs}$. The data as measured show a positive correlation between aU and $^{230}Th_{xs}$. This is an unexpected relationship with no physical basis and the observation of such a relationship in the data is strongly indicative of post depositional dissolution of aU and subsequent under correction for ^{230}Th ingrowth. We expect the correlation between aU and $^{230}Th_{xs}$ in the model to be near zero when we identify a realistic scenario of aU dissolution and daughter isotope 'abandonment'.

In our experiments we varied three parameters: **1)** the hypothesised original aU concentration, **2)** the time elapsed between aU emplacement and dissolution, and **3)** the concentration threshold above which aU is dissolved. To simplify the calculation, we assume that all of the ^{238}U present is authigenic. This is reasonable simplifying assumption because on average the contribution of detrital ^{238}U to the total ^{238}U is ~0.7%. The steps used to generate the hypothetical time series presented in Supplementary Figure 3 can be found in Supplementary Note 2.

Supplementary Figure 3 illustrates two different iterations of the modeling experiment for the same interval depicted in Figure 4. Panels a (solid lines) and b illustrate the positive correlation between aU and $^{230}Th_{xs}$ prior to correction for aU dissolution. Panels a (dotted lines) and c show the results of an experiment in which intervals with an aU concentration above 0.67 ppm undergo dissolution to two-thirds of their initial concentrations 15 ka after the time of aU precipitation. Accounting for this history of aU precipitation and subsequent dissolution would result in the $^{230}Th_{xs}$ time series represented by the dotted blue line. The relationship between the hypothesised $^{230}Th_{xs}$ time series and the observed time series of aU in Panel c shows no correlation (as expected). Panel d depicts the results of an experiment in

which intervals with an aU concentration above 1.1 ppm undergo dissolution to two-thirds of their initial concentrations 20 ka after the time of aU precipitation. Accounting for this history of aU precipitation and subsequent dissolution would result in the $^{230}Th_{xs}$ time series represented by the dotted blue line. The relationship between this hypothetical $^{230}Th_{xs}$ time series and the observed time series of aU (Panel e) shows no correlation (again, as expected).

Our modeling results demonstrate that time series of $^{230}Th_{xs}$ which are uncorrelated with ^{238}U (aU) can be obtained by substituting realistic parameter values into our model. We consider these results an additional line of evidence that aU has been removed from the relict aU peaks at some time after deposition. Based on our experimentation we find it likely that approximately two thirds of the initially precipitated aU was dissolved 15-20 kyr after emplacement leaving behind 'abandoned' ^{230}Th daughter isotopes. This process leads to an overestimate of $^{230}Th_{xs.}$

Evidence from $^{230}Th_{xs}$ that the observed aU peaks represent post depositional dissolution supports our conclusion that aU peaks in 17PC as displayed in Figures 2 and 3 are *in situ* and not the result of post depositional reprecipitation of aU down core. Not only are these peaks most likely original to the sediment intervals in which they are found, the available evidence supports the conclusion that they were initially larger than presently observed.

Supplementary Note 2: Burndown model

Here we describe the steps employed to model burndown. For simplicity we use the same model values as depicted in Supplementary Figure 3a. We use M to denote modeled values (in contrast with observed or analytically determined values), T0 to denote activities at the initial time of aU deposition, T1 to denote activities at the time of aU dissolution/oxidation and T2 to denote activities at present. Note that ^{238}U is mentioned explicitly in this sequence but ^{234}U is also taken into account in the decay equations.

1. Assume all measured concentrations of aU greater than some threshold (0.67 ppm) have undergone burndown
2. Assume initial concentration of aU was higher (2/3 greater) than measured. This quantity is $^{238}U_{M,T0}$ (dotted red line in Supplementary Figure 2)
3. Use equation 1 to estimate the ^{230}Th produced by the decay of $^{238}U_{M,T0}$ after some number of years (T1=15,000 yrs), this is $^{230}Th_{M,T1}$

$$^{230}Th_{M,T1} = ^{238}U_{M,T0} *$$
$$\left[(1 - e^{-\lambda_{230} * T1}) + \frac{\lambda_{230}}{\lambda_{230} - \lambda_{234}} * (e^{-\lambda_{234} * T1} - e^{-\lambda_{230} * T1})(\frac{^{234}U}{^{238}U})_{init} \right] \quad (1)$$

4. Calculate the ^{238}U left after some period of anoxia in the sediments (15,000), this is the decay corrected $^{238}U_{M,T0}$ (The decay is very small due to the long half-life of ^{238}U)
5. Assume that aU dissolution (2/3) occurs and multiply $^{238}U_{M,T0}$ by this fraction. The resulting quantity is $^{238}U_{M,T1}$
6. Use equation 2 to calculate the ^{230}Th produced by $^{238}U_{M,T1}$ decay during the remainder of the accumulation time (T2 = T0-T1), this is $^{230}Th_{M,T2}$

$$^{230}Th_{M,T2} = ^{238}U_{M,T1} *$$
$$\left[(1 - e^{-\lambda_{230} * T2}) + \frac{\lambda_{230}}{\lambda_{230} - \lambda_{234}} * (e^{-\lambda_{234} * T2} - e^{-\lambda_{230} * T2})(\frac{^{234}U}{^{238}U})_{init} \right] \quad (2)$$

7. Subtract the sum of $^{230}Th_{M,T1}$ and $^{230}Th_{M,T2}$ from the observed/measured ^{230}Th to yield $^{230}Th_{M,xs}$
8. $^{230}Th_{M,xs}$ is the modeled/hypothesized $^{230}Th_{xs}$ given the scenario of dissolution outlined by the model parameters (dotted blue line in Supplementary Figure 2)

Supplementary Figure 1. Age-Depth Relationship in Core 17PC

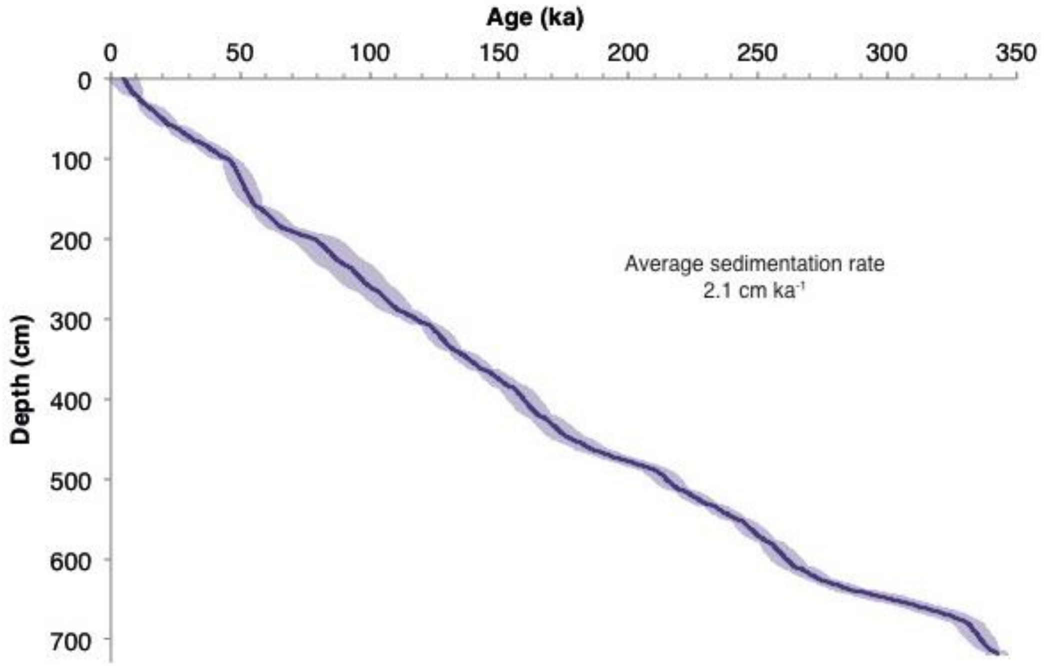

Data for core ML1208-17PC (purple line) with shading indicating two sigma uncertainty (95% confidence intervals) for the age model. The accumulation rate depicted on the figure is the average for the 350 kyr interval discussed here.

Supplementary Figure 2. Episodic co-variation of $^{230}Th_{xs,0}$ and aU

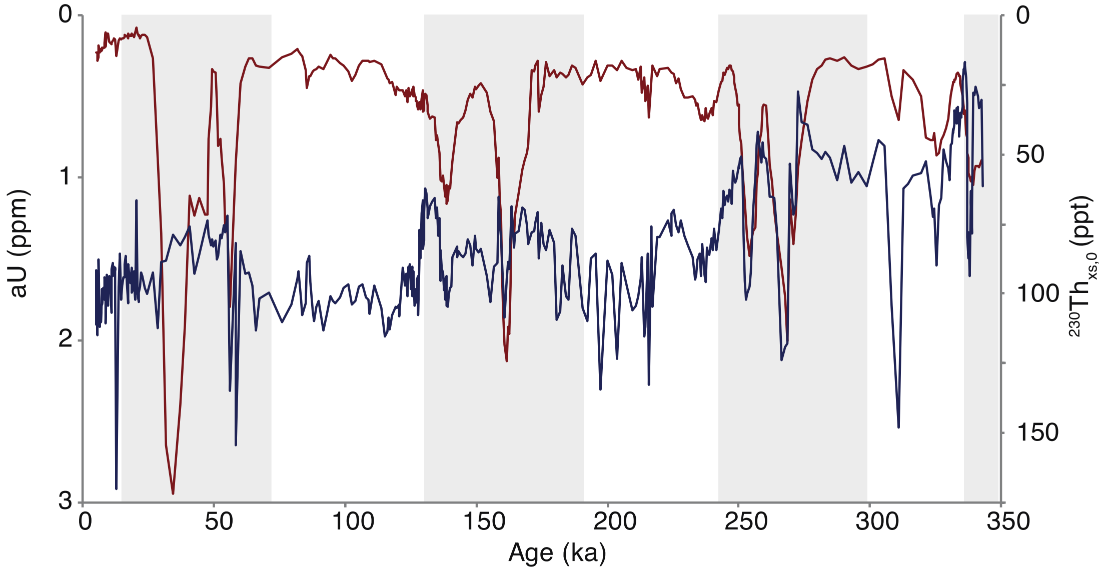

Records of aU (red) and $^{230}Th_{xs,0}$ (blue) from site 17PC over the last 350 kyr. Note areas of correlation between aU and $^{230}Th_{xs,0}$ within glacial intervals (grey bars).

Supplementary Figure 3. Results of aU Modeling Exercise

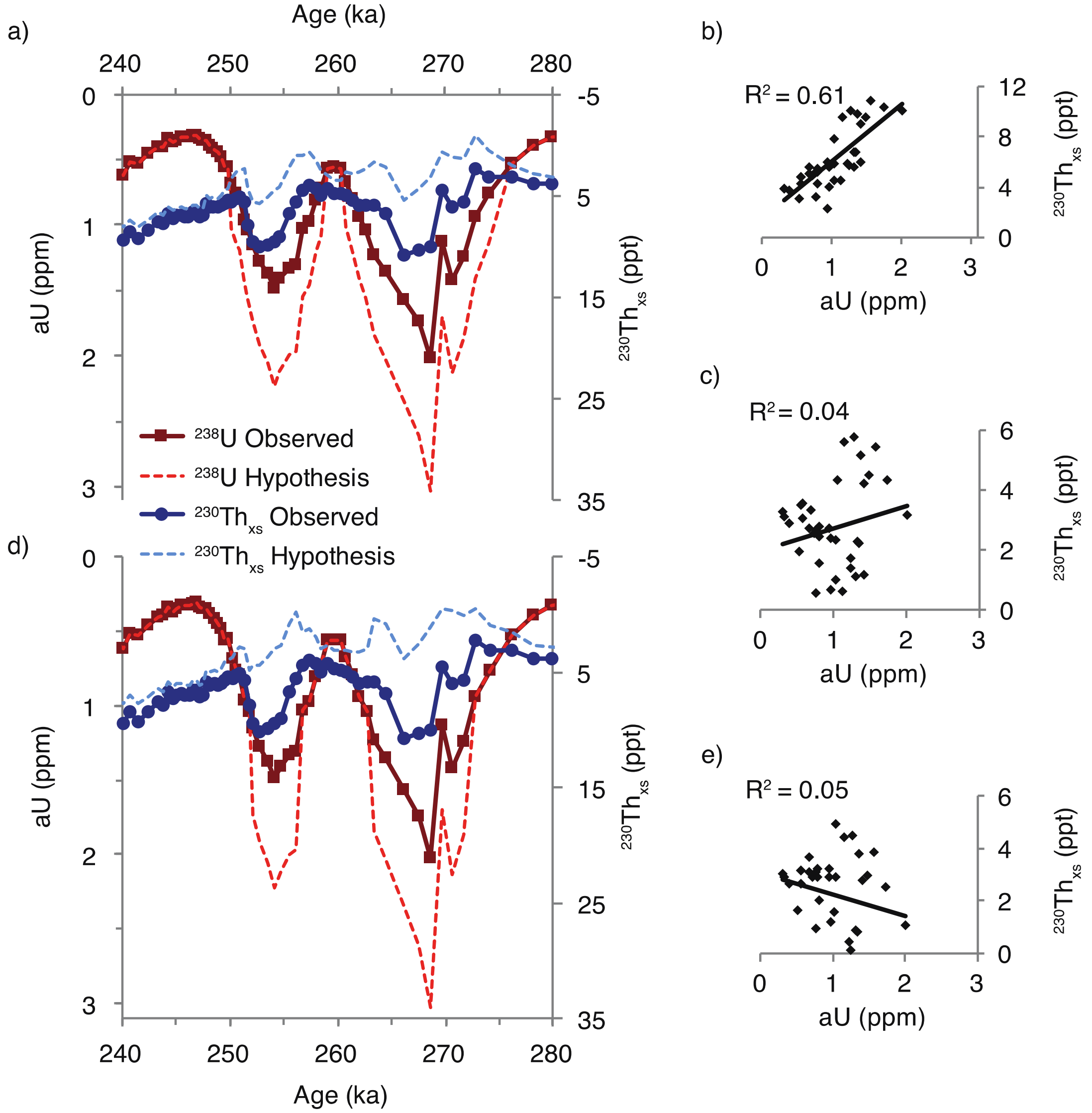

Here we show two different scenarios of post-depositional aU dissolution. Panel **(a)** shows the results of an experiment in which intervals with an aU concentration above 0.67 ppm undergo dissolution to two-thirds of their initial concentrations, 15 ka after the time of aU precipitation. Panel **(b)** shows the observed correlation between $^{230}Th_{xs}$ and aU in the data as measured. Panel **(c)** shows the same correlation but for the hypothetical $^{230}Th_{xs}$ and aU data. Panel **(d)** depicts the results of an experiment in which intervals with an aU concentration above 1.1 ppm undergo dissolution to two-thirds of their initial concentrations 20 ka after the time of aU precipitation. Panel **(e)** shows the correlation between hypothetical $^{230}Th_{xs}$ and aU data. Panels a and b the observed aU (maroon) and $^{230}Th_{xs}$ (navy) data are displayed with solid lines and symbols and the hypothesised original values of aU (red) and $^{230}Th_{xs}$ (blue) are shown with dotted lines.

Supplementary Table 1. Cores referenced in this work.

Name	Latitude (DDS)	Longitude (DDS)	Depth (m)	Reference
ML1208-37BB	7.0	-161.6	2798	This study and Jacobel *et al.*, 2017[1]
ML1208-31BB	4.9	-160.1	2857	This study and Jacobel *et al.*, 2017[1]
ML1208-17PC	0.5	-156.5	2926	This study and Jacobel *et al.*, 2017[1]
TTN013-18	-2.0	-140.0	4400	Broecker and Clark, 2010[2]
TT013-PC72	0.1	-139.4	4300	Anderson *et al.*, 2006[3], Winckler *et al.*, 2008[4]
KNR73 6PG	-1.6	-114.2	3806	Keigwin *et al.*, 2015[5]
ODP 849	0.2	-110.5	3851	Pichat *et al.*, 2004[6]
KNR73 4PC	4.0	-110.3	3681	Keigwin *et al.*, 2015[5]
KNR73 3PC	-0.4	-106.2	3606	Keigwin *et al.*, 2015[5]
PLDS 7G	-3.3	-102.5	3253	Keigwin *et al.*, 2015[5]
VNTR01 10 GC	-4.5	-102.0	3410	Keigwin *et al.*, 2015[5]
GS7202-15	3.3	-97.9	2986	Keigwin *et al.*, 2015[5]
RC13-140	2.9	-87.8	2246	Bradtmiller *et al.*, 2010[7]
RC11-238	-1.5	-85.8	2573	Bradtmiller *et al.*, 2010[7]
MD97-2138	-1.2	146.2	1900	Bradtmiller *et al.*, 2010[7]
S67 15FFC	-1.6	162.6	4250	Keigwin *et al.*, 2015[5]
ODP 882	50.4	167.6	3244	Jaccard *et al.*, 2009[8]

Supplementary References

1. Jacobel, A. W., McManus, J. F., Anderson, R. F. & Winckler, G. Climate-related response of dust flux to the central equatorial Pacific over the past 150 kyr. *Earth and Planetary Science Letters* **457,** 160–172 (2017).
2. Broecker, W. S. & Clark, E. Search for a glacial-age ^{14}C-depleted ocean reservoir. *Geophysical Research Letters* **37,** DOI: 10.1029–2010GL043969 (2010).
3. Anderson, R., Fleisher, M. & Lao, Y. Glacial–interglacial variability in the delivery of dust to the central equatorial Pacific Ocean. *Earth and Planetary Science Letters* **242,** 406–414 (2006).
4. Winckler, G., Anderson, R. F., Fleisher, M. Q., McGee, D. & Mahowald, N. Covariant Glacial-Interglacial Dust Fluxes in the Equatorial Pacific and Antarctica. *Science* **320,** 93–96 (2008).
5. Keigwin, L. D. & Lehman, S. J. Radiocarbon evidence for a possible abyssal front near 3.1 km in the glacial equatorial Pacific Ocean. *Earth and Planetary Science Letters* **425,** 93–104 (2015).
6. Pichat, S. *et al.* Lower export production during glacial periods in the equatorial Pacific derived from $(^{231}Pa/^{230}Th)_{xs,0}$ measurements in deep-sea sediments. *Paleoceanography* **19,** DOI: 10.1029–2003pa000994 (2004).
7. Bradtmiller, L. I., Anderson, R. F., Sachs, J. P. & Fleisher, M. Q. A deeper respired carbon pool in the glacial equatorial Pacific Ocean. *Earth and Planetary Science Letters* **299,** 417–425 (2010).
8. Jaccard, S. L., Galbraith, E. D., Sigman, D. M. & Haug, G. H. A pervasive link between Antarctic ice core and subarctic Pacific sediment records over the past 800kyrs. *Quaternary Science Reviews* **29,** 206–212 (2010).
