# Supplementary material for: Repeated storage of respired carbon in the equatorial Pacific Ocean over the last three glacial cycles
Source: Nat Commun. 2017 Nov 23;8:1727. doi: 10.1038/s41467-017-01938-x (PMC5700088; doi:10.1038/s41467-017-01938-x)
Supplement: Supplementary file 2 — Description of Additional Supplementary Files [file 41467_2017_1938_MOESM2_ESM.pdf]

Description of Additional Supplementary Files

File Name: Supplementary Data 1
Description: U-series data for ML1208 cores. New data from ML1208 sediment cores 17PC, 31BB and 37BB including age-depth relationships, aU concentration data and $^{230}Th_{xs,0}$ activity data. Uncertainties are reported at the 1 s.d. level.
